# Melioidosis in dogs and cats: 18 cases

**DOI:** 10.1093/jvimsj/aalag221

**Published:** 2026-09-30

**Authors:** Ziyue Sharon Lim, Kent Soon, Nicholas Neo, Jose Labrador, Darren Kelly

**Affiliations:** Internal Medicine Department, VES Hospital, 232 Whitley Road, 297824, Singapore; Internal Medicine Department, VES Hospital, 232 Whitley Road, 297824, Singapore; Internal Medicine Department, VES Hospital, 232 Whitley Road, 297824, Singapore; Diagnostic Imaging Department, VES Hospital, 232 Whitley Road, 297824, Singapore; Internal Medicine Department, VES Hospital, 232 Whitley Road, 297824, Singapore

**Keywords:** *Burkholderia pseudomallei*, cutaneous abscess, melioidosis, prostatitis, prostatic abscess, osteomyelitis

## Abstract

**Background:**

The clinical manifestations and outcomes in dogs and cats with melioidosis have not been described.

**Hypothesis/Objectives:**

Describe the presenting signs, treatment, and outcome of dogs and cats with melioidosis.

**Animals:**

Fifteen dogs and 3 cats from a hospital in Singapore.

**Methods:**

Retrospective case series. A hospital database search was performed to identify dogs with melioidosis presenting over a 5-year period. Data collected included signalment, clinical signs, test results, treatment, and outcome.

**Results:**

Diagnosis was based on positive bacterial culture and identification of *Burkholderia pseudomallei* in all cases. At presentation, 13/15 (87%) had systemic inflammatory response syndrome (SIRS), 10/15 (67%) were septic, and 3/15 (20%) had septic shock. In dogs, 8/15 (53%) had multiple cutaneous abscesses or open wounds, and 6/15 (40%) had prostatitis or prostatic abscessation. In cats, 2/3 had osteomyelitis and 1/3 had pyelonephritis. Of the cases, 5/15 (33%) dogs and none of the cats were considered potentially immunocompromised. Of the 17 cases with >4 weeks of follow-up, death or euthanasia attributed to melioidosis occurred in 8/17 (47%). Of the surviving 9/17 (53%) animals, 6 were alive after 1 month (range, 1-43 months) and 3 died or were euthanized for reasons unrelated to melioidosis, with median follow-up duration of 724 days.

**Conclusions and clinical importance:**

Melioidosis should be considered in dogs with prostatitis or cutaneous abscesses, and in cats with osteomyelitis. Initial treatment with ceftazidime or meropenem, followed by 3-6 months of trimethoprim-sulfonamide (TMPS) might be optimal.

## Introduction

Melioidosis is caused by the aerobic, gram-negative, mobile bacillus, *Burkholderia pseudomallei,* an environmental saprophyte with most cases occurring in tropical and subtropical regions. Although most commonly reported in Southeast Asia and Northern Australia, ^1–5^ the bacterium is considered endemic in at least 45 countries, with the Gulf Coast region of the United States recently declared an endemic region by the Centers for Disease Control and Prevention (CDC; 6-8). There are concerns regarding the possibility of spread to regions currently not considered endemic, and sporadic reports in non-endemic regions suggest that unrecognized foci might already exist.^2^ With the increasing movement of humans and animals across the globe, there is a risk of infected pets being imported from endemic regions to non-endemic regions, and awareness and an understanding of the bacterium and associated disease are important for veterinary staff dealing with pets imported from endemic regions. *B pseudomallei* is also considered a potential bioterrorism agent and, considering the potentially serious risks the bacterium poses to public health, a published report described the public health response to an infected dog imported into New York from Thailand.^9^

In endemic regions, *B pseudomallei* can be isolated from moist soil and muddy water, its primary reservoirs.^1–5^ The bacterium is found in higher numbers in deeper soil layers, and periods of rainfall result in percolation of the bacterium to the surface, increasing contact risk.^2–4^^,^^8^ Outbreaks have been associated with soil disruption such as with redevelopment work, after flooding, or with landform degradation and geological events.^3^^,^^8^ Transmission is usually by direct contact with contaminated soil or water, either by inhalation, ingestion, or percutaneous inoculation and exposure of open wounds.^1–5^ Clinical presentation varies depending on species, site of infection, stage of disease, and even among different geographical regions.^2^^,^^3^^,^^5^ In Singapore, the most common presentations in humans are bacteremia (60.3%), deep organ abscesses (40.7%), and pneumonia (33.1%; 1,2). Asymptomatic infection also appears relatively common, and reactivation after long periods of dormancy is also a characteristic of melioidosis in humans.^2^ Similar situations also might be true in dogs because the only existing, recently published serological study, which included 156 shelter dogs in Thailand, found that 9/156 (6%) were seropositive for *B pseudomallei.*^10^

Melioidosis affects a wide range of species, including cats, dogs, sheep, goats, horses, primates, deer, elephants, cattle, camels, crocodiles, zebras, iguanas and avian species.^1^^,^^3^^,^^5^ The limited published data describing melioidosis in dogs and cats consists primarily of case reports. Currently, only 1 report of a dog in Australia,^11^ 1 report of a dog in Thailand,^12^ and a case series describing only the histopathologic changes in 7 dogs diagnosed while serving with the United States Armed Forces in Vietnam in 1970^13^ have been published. Regarding cats, 1 report of a cat in Malaysia,^14^ 1 report of a cat in New Caledonia,^15^ and 2 reports each of 2 cats in Australia^16^^,^^17^ have been described. Reported signs in dogs and cats are variable and include fever, weight loss, lethargy, cutaneous abscesses, abscessation of internal organs, pneumonia, and septicemia.^11–17^

We describe the clinical aspects of melioidosis in a population of dogs and cats from Singapore, with the aim of increasing awareness of the disease to facilitate earlier diagnosis and institution of appropriate treatment in suspected cases.

## Materials and methods

Ethical approval was granted by Mars Veterinary Health Veterinary Clinical Studies Committee. The electronic medical database of a single emergency and specialist referral hospital in Singapore was searched to identify client-owned dogs and cats diagnosed with melioidosis between May 2020 and July 2025. Search terms used were “Burkholderia”, “pseudomallei”, and “melioidosis”. Dogs and cats were eligible for inclusion if a positive culture with bacterial identification or positive PCR for *B pseudomallei* was available. Cases were excluded if the diagnosis was suspected but without confirmation by culture or PCR. All cases meeting the inclusion criteria were included.

Data collected from the medical records included species, breed, sex, neuter status, age at presentation, concurrent or pre-existing diseases, clinical signs, duration of signs, diagnostic test results, treatment before presentation, culture or PCR results, treatment, and case outcome. When available, results of other diagnostic tests were recorded, including hematology, biochemistry, urinalysis, and diagnostic imaging. All case data were reviewed by a board-certified diplomate in internal medicine, and all thoracic radiographs were reviewed by a board-certified diplomate in diagnostic imaging.

For dogs, a diagnosis of systemic inflammatory response syndrome (SIRS) was made if ≥2 of the following criteria were met; temperature ≥39.7 °C or ≤ 37.8 °C, heart rate ≥160/min, respiratory rate ≥40/min, and white blood cell count ≥12 × 10^9^/L or ≤4 × 10^9^/L or ≥10% bands. Sepsis was defined as the presence of infection and those meeting the aforementioned criteria for SIRS (also considered the “Sepsis-2” criteria), and the presence of organ dysfunction defined as a Sequential Organ Failure Assessment score ≥2.^18^^,^^19^ Septic shock was defined as systolic blood pressure ≤90 mm Hg (or mean blood pressure <65 mm Hg) despite adequate hydration and necessitating vasopressor support and a blood lactate concentration >2 mmol/.^19^

## Results

Fifteen dogs and 3 cats met the inclusion criteria. In all cases, the diagnosis was based on positive bacterial culture of *B pseudomallei* and identification by means of matrix-assisted laser desorption/ionization—time-of-flight mass spectrometry (Antech Veterinary Diagnostics, Singapore). Diagnosis was not based on serology or PCR in any case.

### Signalment

Of the 15 included dogs, 7/15 (47%) were intact males, 4/15 (27%) were neutered males, 3/15 (20%) were neutered females, and 1/15 (7%) were intact females. Of the 3 included cats, 2 were neutered males, and 1 was a neutered female. Median age at presentation was 7.4 years (range, 2.3-12 years). Of the 15 dogs, 4 were cross breeds, 2 were Rottweilers, and 1 each was of the following breeds: Boxer, Dutch Shepherd dog, Chow Chow, Shetland sheepdog, Labrador retriever, English Springer spaniel, Miniature schnauzer, Shih Tzu, and Shiba Inu. Of the 3 cats, 2 were domestic shorthair, and 1 was a Persian cross.

### Antimicrobials before presentation

Of the included dogs, 6/15 were receiving antimicrobials at presentation. These included amoxicillin-clavulanate alone in 4, amoxicillin-clavulanate and metronidazole in 1, and an unspecified antimicrobial in 1. All 3 affected cats were receiving antimicrobials at presentation, with 1 each receiving amoxicillin-clavulanate, doxycycline, and clindamycin.

### Clinical signs and diagnoses (dogs)

Median duration of signs before diagnosis was 13 days (range, 1-60 days). Pyrexia (rectal temperature >39.3 °C for dogs) was documented on at least 1 occasion in 14/15 (93%) dogs. At presentation, 13/15 (87%) dogs met the criteria for SIRS and 10/15 (67%) met the criteria for sepsis. Of the included dogs, 3/15 (20%) presented in septic shock or developed septic shock during hospitalization.

Of the included dogs, 8/15 (53%) presented with 1 or multiple cutaneous abscesses or open wounds (Figure 1). Of these, 1 was also diagnosed with prostatitis, and 1 was also diagnosed with pyothorax, multiple lung abscesses, and abscessation of the adrenal glands, mesenteric lymph nodes, and possibly liver. Of the included dogs, 6/15 (40%) were diagnosed with prostatitis or prostatic abscessation. Of these, 5/6 also had changes consistent with septic peritonitis based on ultrasonographic findings, including the presence of echogenic abdominal effusion and hyperechoic mesentery, with 3 being confirmed based on fluid analysis showing neutrophilic inflammation with intracellular bacteria, and no fluid analysis was performed in the other 2. Of the included intact male dogs, 6/7 were diagnosed with prostatitis or prostatic abscessation, and the remaining dog lacked diagnostic test results to determine if prostatic disease was present. Of the remaining included dogs, 1 was diagnosed with mastitis post-whelping, and 1 (a neutered male) was diagnosed with bacterial urinary tract infection with no evidence of prostatitis on abdominal ultrasonography.

**Figure 1:**
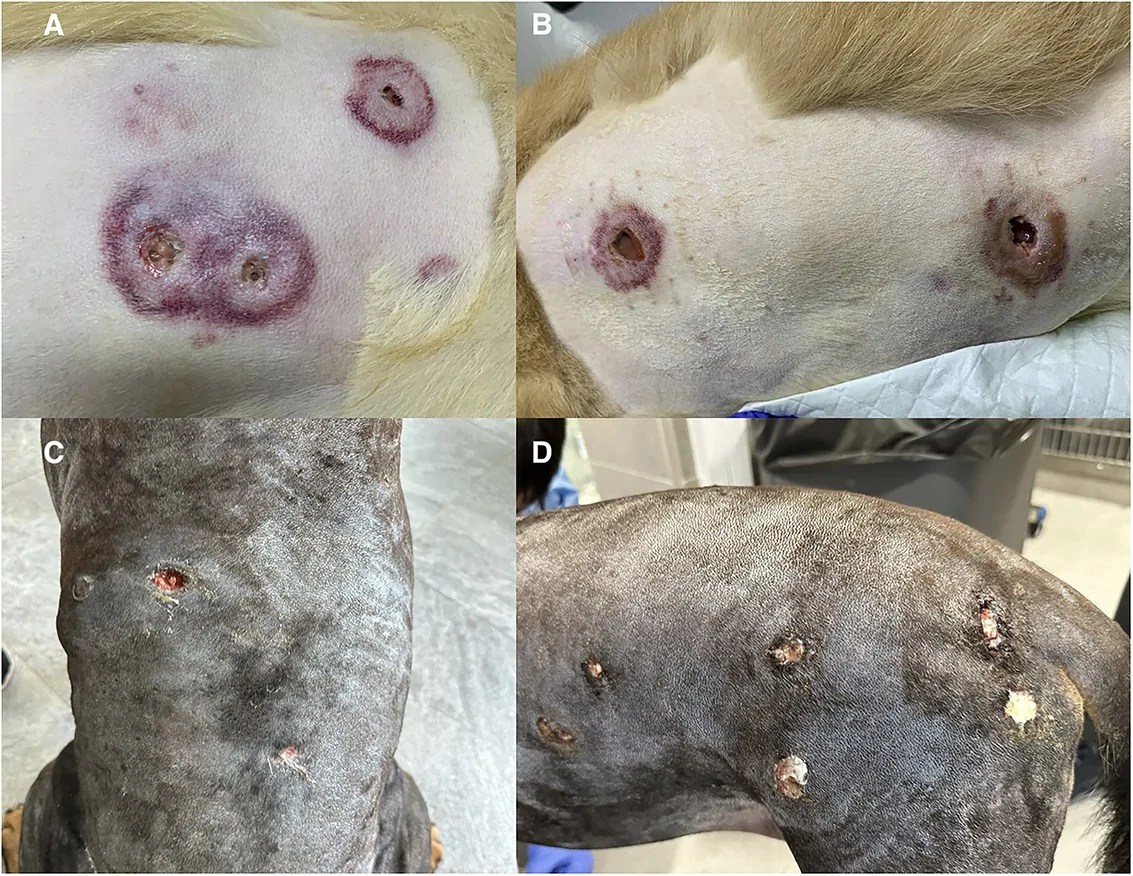
Photos of 2 dogs (A and B from 1 dog, and C and D from another dog) with multiple cutaneous abscesses and open wounds. Melioidosis was confirmed in both cases based on culture of *Burkholderia pseudomallei* from the lesions.

### Clinical signs and diagnoses (cats)

Pyrexia (rectal temperature >39.5 °C for cats) was documented on at least 1 occasion in 3/3 cats. The ages of the cats were 11, 60, and 77 months. Of the 3 cats, 1 was diagnosed with pyelonephritis caused by *B pseudomallei* based on ultrasonographic findings consistent with severe bilateral pyelonephritis and positive urine culture. One cat was diagnosed with osteomyelitis of the right humerus based on culture from a swab taken from the medullary cavity of the affected bone. This cat had computed tomography (CT) findings consistent with osteomyelitis of the right humerus (Figure 2) and histopathology consistent with suppurative osteomyelitis. One was diagnosed with osteomyelitis of the bones of the left tarsus and associated septic arthritis of the tarsal joint based on culture from a sample of joint fluid. This cat had CT findings consistent with osteomyelitis affecting multiple bones of the left tarsus.

**Figure 2:**
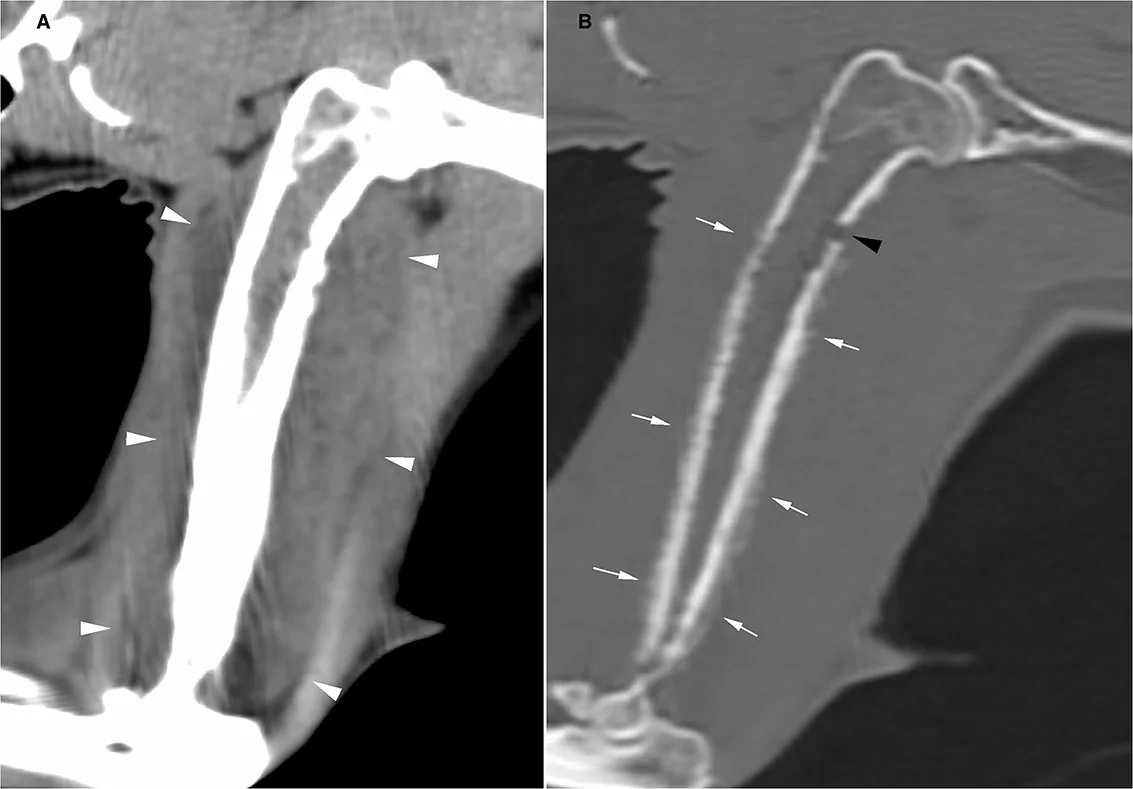
Native CT reformatted sagittal reconstruction images of the right humerus of the cat diagnosed with osteomyelitis of this bone, displayed in a soft tissue window (A) and bone window (B). A large hypoattenuating soft tissue swelling is noted extending along the length of the brachium surrounding the humerus (white arrow heads). Extensive irregular/interrupted periosteal reaction is noted along the cranial and caudal cortical margins (white arrows) with associated permeative to moth-eaten cortical lysis and areas of cortical interruption (black arrowhead).

### Hematology and biochemistry

Of the included dogs, 11/15 had a neutrophilia, 3/15 had neutropenia, and 1/15 had a neutrophil count within reference range. Mild, non-regenerative anemia was documented in 6/15 dogs, and thrombocytopenia of variable severity was documented in 6/15. Two of the 3 included cats had mild neutrophilia, and 1 had mild neutropenia. One cat had mild, non-regenerative anemia. Hyperglobulinemia was documented in 10/15 dogs and in 1/3 cats. Mildly increased activity of 1 or more hepatobiliary enzymes was documented in 6/15 dogs and in 1/3 cats. Two dogs had hypoglycemia at presentation (both with septic shock), and 2 dogs had mild hyperbilirubinemia (both with sepsis).

### Sample types

In 7/8 dogs presenting with cutaneous abscesses or open wounds, the sample type that returned a positive culture for *B pseudomallei* was fluid from an abscess or a swab from an open wound, and in 1 dog that had both cutaneous abscesses and prostatitis, the positive culture was from urine. Of the 6 dogs with prostatitis or prostatic abscessation, the sample type that returned a positive culture for *B pseudomallei* was urine in 2/6, prostatic abscess fluid in 2/6, prostatic wash fluid in 1/6, and abdominal effusion in 1/6. In 2 of these dogs that returned positive culture results on samples from the prostate, more than 1 sample type was submitted for bacterial culture, and in both, positive cultures for *B pseudomallei* were obtained from both urine and abdominal fluid. In the dog with septic mastitis, the positive culture was from a swab of purulent material from the infected gland, and in 1 neutered male dog presenting with lethargy, anorexia, and fever, and with no focus of infection identified on physical examination or thoracic and abdominal imaging, the sample type from which positive culture was obtained was urine. Antimicrobial susceptibility testing was not performed on any isolate in accordance with the laboratory’s protocols, because *B pseudomallei* is considered a Biosafety Level 3 pathogen.

### Diagnostic imaging

Thoracic imaging was performed in 8/15 dogs, with all 8 having thoracic radiography and 2 having thoracic radiography and CT. Of these 8 dogs, 3/8 (38%) had changes consistent with multiple lung abscesses (Figure 3) and each of these dogs presented primarily with multiple cutaneous abscesses. Complete abdominal imaging was performed in 8/15 dogs, with an additional 2 (both with prostatitis or prostatic abscessation) having only focused ultrasonography of the urinary system performed. Of the 8 dogs that had complete abdominal imaging, 7 had abdominal ultrasonography, and 1 had CT performed. Of these 8 dogs, 4 were diagnosed with prostatitis or prostatic abscessation, 1 with multiple cutaneous abscesses, 1 with pyothorax and multiple lung abscesses, 1 with mastitis, and 1 with urinary tract infection. Of these 8 dogs, imaging changes consistent with infection or abscessation of any organ aside from the prostate was only identified in 1 dog, which had pyothorax and multiple lung abscesses and CT changes consistent with bilateral adrenal gland abscesses, abscessation of multiple mesenteric lymph nodes, and possible early multifocal hepatic abscessation.

**Figure 3:**
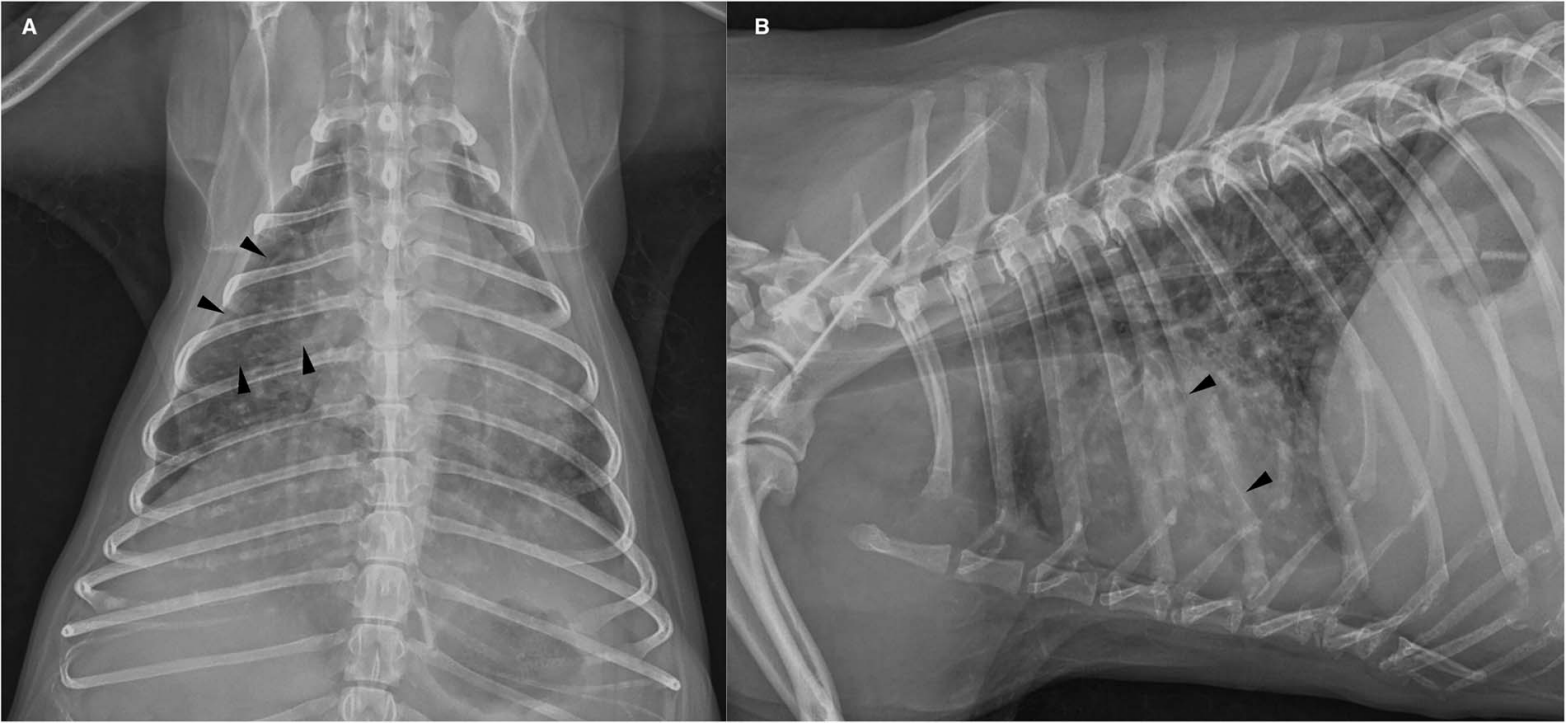
Dorsoventral (A) and right lateral (B) radiographs of the thorax of a dog with melioidosis and presumed multiple lung abscesses, demonstrating a diffuse bronchointerstitial lung pattern with multifocal nodular opacities. Thin pleural fissure lines are indicated by the black arrowheads.

### Pre-existing or concurrent diseases or medications that could result in immune compromise

Of the 3 cats, 2 were tested for feline immunodeficiency virus (FIV) and feline leukemia virus (FeLV; SNAP FIV/FeLV Combo Test, IDEXX) at presentation and were negative for both, and the third was not tested. No other pre-existing or current immunosuppressive condition was identified in any of the cats. Of the included dogs, 5/15 (33%) were receiving medications or had a disease or physiologic state considered potentially immune-compromising, with 2 receiving immunosuppressive doses of prednisolone (1 for chronic inflammatory enteropathy and 1 for sterile pyogranulomatous lymphadenitis), 1 receiving oclacitinib for atopy, 1 had diabetes mellitus, and 1 had recently whelped.

### Surgical intervention

In 2 dogs, both with single large cutaneous abscesses, surgical drainage and irrigation of the abscess was performed. The other 6 dogs with cutaneous manifestations had either multiple abscesses or open wounds, and surgical intervention was not performed. In 4/6 dogs with prostatitis, surgical intervention was performed for abscess drainage, omentalization, and abdominal lavage. Surgical intervention was not performed in 2/6 dogs with prostatitis; 1 that had diffuse prostatic changes on ultrasonography with abdominal effusion but without overt abscess formation, and 1 that had diffuse prostatic changes on ultrasonography and multiple cutaneous abscesses. Mastectomy was performed in the dog with mastitis. Surgical debridement and irrigation were performed in the cat with osteomyelitis of the right humerus.

### Initial antimicrobial choice

Of the 18 animals, the antimicrobials initially prescribed on presentation and before culture of *B pseudomallei* were amoxicillin-clavulanate and a fluoroquinolone in 11, amoxicillin-clavulanate alone in 3, clindamycin and a fluoroquinolone in 2, doxycycline alone in 1, and trimethoprim-sulfonamide (TMPS) and meropenem in 1. Of the 18 animals, 4/18 died or were euthanized before culture results were available and, of these, 1 was receiving amoxicillin-clavulanate and marbofloxacin, 1 was receiving clindamycin and marbofloxacin, 1 was receiving amoxicillin-clavulanate alone, and 1 was receiving meropenem and TMPS. All 4 of these dogs were septic, with 2 presenting in septic shock.

### Subsequent antimicrobial choice

Of the 14 animals alive at the time positive culture for *B pseudomallei* was received, the initial antimicrobial treatment was changed in 7/14 (50%), with 3 changed to TMPS alone, 1 changed to ceftazidime and TMPS, 1 changed to meropenem and TMPS, 1 changed to meropenem alone, and 1 changed to doxycycline and TMPS. Of the 12/18 animals discharged from the hospital, 5/12 were discharged with TMPS alone, 3/12 with amoxicillin-clavulanate and a fluoroquinolone, 2/12 with amoxicillin-clavulanate alone, 1/12 with doxycycline alone, and 1/12 with clindamycin and enrofloxacin. Of the 12 animals discharged from the hospital, the cat with osteomyelitis of the left humerus died 22 days after discharge while on treatment with amoxicillin-clavulanate and enrofloxacin; 1 was alive with <3 months of follow-up and with a plan to complete a 20-week course of TMPS, 1 was lost to follow-up after 15 days but had been discharged with 4 weeks of amoxicillin-clavulanate and enrofloxacin, and >6 months follow-up was available for 9 cases. Of the 9 cases discharged from the hospital and with long-term follow-up, the median duration of antimicrobial treatment was 16 weeks (range, 4-24 weeks). The type, duration, and dose of the antimicrobials used are presented in Supplementary Item 1.

### Outcomes and survival based on dog physiologic status at presentation

Of the 18 cases, 3/18 dogs presented in septic shock and these 3 dogs died or were euthanized in the hospital. Of the 18 cases, 8/18 met the criteria for sepsis without septic shock. Of these, 5/8 survived to discharge, with 1 lost to follow-up <4 weeks after discharge and 1 died <4 weeks after discharge, and 3/8 were euthanized in the hospital. Therefore, of the 11/18 dogs that presented with sepsis, with or without septic shock at presentation, only 5/11 survived to discharge, and only 3/10 (censoring for the case lost to follow-up) survived >4 weeks after discharge. Of the 18 animals, 5/18 presented with SIRS without sepsis and 2/18 presented without SIRS. Of these, 7/18 animals that presented without SIRS or with SIRS without sepsis all 7 survived to discharge and for >4 weeks afterward.

### Overall outcomes and survival

Of the 18 animals, 12/18 (67%) were discharged and 6/18 (33%) died or were euthanized during hospitalization. Of the 12 animals discharged, 1 was alive but lost to follow-up after 2 weeks, 1 died <4 weeks after discharge because of melioidosis, and 10 were alive with >4 weeks of follow-up available. With the exclusion of 1 case from long-term survival analysis because of <4 weeks follow-up being available, death or euthanasia attributed to melioidosis occurred in 8/17 (47%) cases, with 6 animals not surviving hospitalization, 1 dying <4 weeks after discharge, and 1 euthanized 515 days after diagnosis because of recurrence of signs. Of the other 9/17 (53%) animals, 6 were alive 1 month after discharge (range, 1-43 months) and 3 died or were euthanized for reasons unrelated to melioidosis. Excluding the case discharged and then lost to follow-up within 4 weeks, 9/17 (53%) survived for >4 weeks with no recurrence of signs, with a median follow-up duration of 724 days (mean, 534 days; range, 36-1277). Case outcomes are described in Supplementary Item 1.

### Spatial and temporal distributions

Figure 4 presents the spatial distribution of the 18 cases based on where they primarily resided. The month in which each dog was first presented for veterinary attention for clinical signs attributable to melioidosis is presented in Supplementary Item 1. Figure 5 presents the average monthly rainfall for Singapore and the number of cases initially presenting in each month.

**Figure 4:**
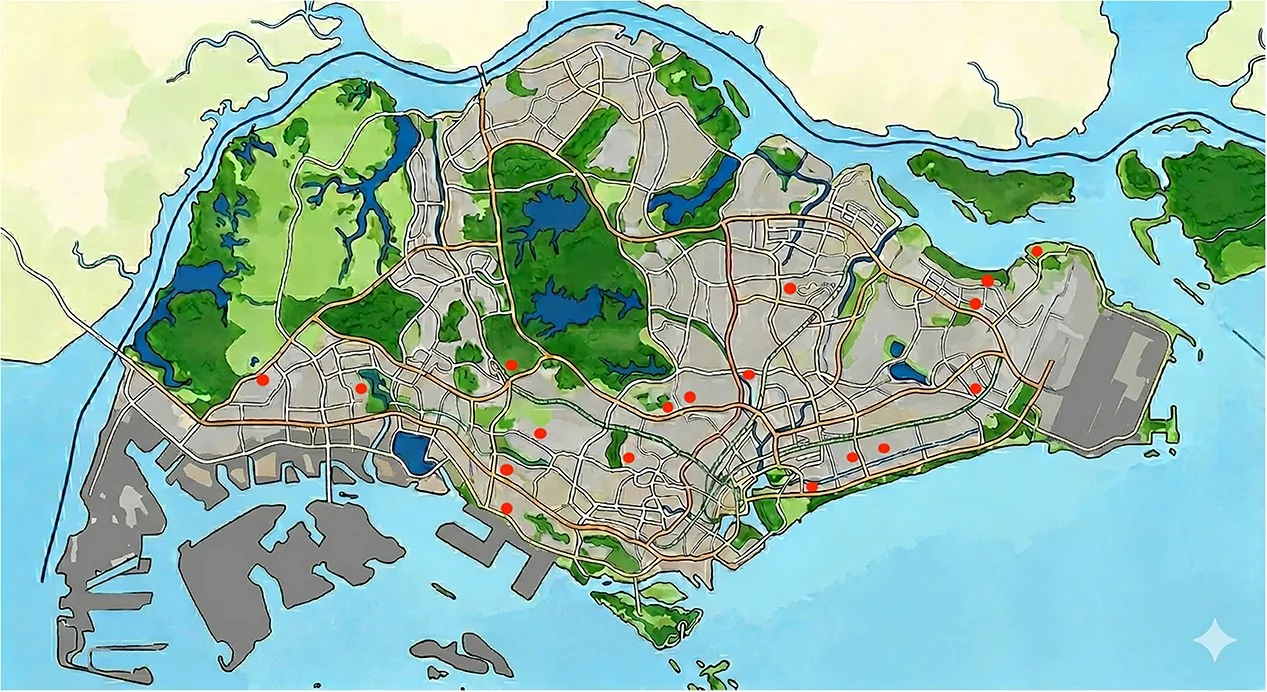
Stylized map of Singapore demonstrating the distribution of the 18 included cases. Each dot indicates the location in which one affected animal primarily resided.

**Figure 5:**
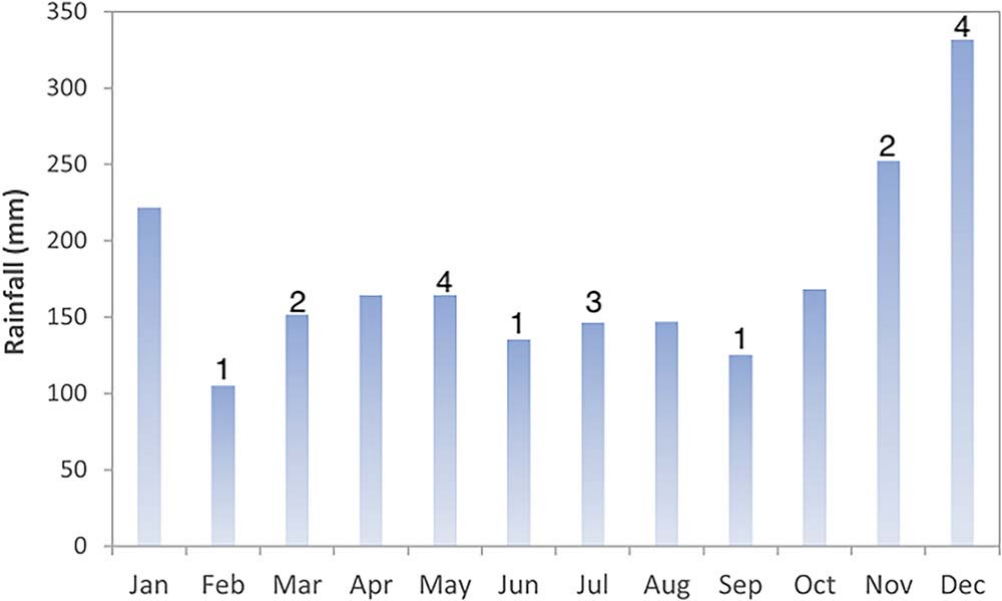
Graph modified from Meteorological Service of Singapore (https://www.weather.gov.sg/climate-climate-of-singapore/) detailing the average monthly rainfall in Singapore between 1991 and 2020. The number appearing on top of each bar is the number of cases, from the 18 included in our study, initially presenting during that month.

## Discussion

Although melioidosis has been recognized in humans since 1920, it has been under-reported in domestic animals.^1–3^ Our findings suggest that the presentation in dogs and cats resembles the multisystemic presentation in humans, and that cutaneous abscessation (53% of included dogs) and prostatic involvement (40% of included dogs and 85% when considering only the included intact male dogs) are the hallmark features in this population. Genitourinary and cutaneous manifestations are the second and third most common presentations in humans, occurring in 14% and 13% of cases, respectively.^20^ Because heavy rain increases exposure to *B pseudomallei*,^2–4^^,^^8^ meteorological and spatial patterns of infection were evaluated. No monthly or seasonal patterns were identified. This finding might be because, although monthly variations in rainfall exist, it rains on average 171 days of the year in Singapore. Alternatively, a prolonged or variable incubation period might exist in dogs and cats as in humans.^2^ No subjectively obvious spatial clustering of cases was observed. Based on the geography of the country, it is not surprising that an obvious spatial cluster of disease was not identified because there is easy and close access to rural and green areas for dog walking and other recreational activities, regardless of where one might primarily reside on the island.

Melioidosis risk factors in humans include male sex, older age, immunosuppression (eg, corticosteroid administration), and comorbidities such as diabetes mellitus and chronic granulomatous disease.^1^^,^^2^^,^^4^^,^^8^^,^^20^ Human cases in Singapore spanned 1 month-97 years, peaking at ≥45 years of age with a 4.5:1 male-to-female ratio.^1^^,^^4^ Similarly, dogs showed a 73% male predisposition.^1^^,^^4^^,^^21^ Up to 77% of affected humans have comorbidities, primarily diabetes mellitus (37%-60%; 2,21,22). Although only 1 dog had diabetes, 33% were potentially immunocompromised because of medications or physiological state, with 2 receiving immunosuppressive dosages of corticosteroids (1 for pyogranulomatous lymphadenitis) and 1 received oclacitinib. Thus, although healthy animals are susceptible, immune dysfunction may predispose dogs to melioidosis or influence its pathogenesis.

A key finding is the high prevalence of prostatic infection: 85% of affected intact male dogs had prostatitis or prostatic abscessation. Unreported in veterinary literature, this finding mirrors melioidosis in humans, where prostatic abscesses affect 18%-20% of male patients.^2^^,^^20^^,^^22–24^ This observation is relevant for 2 reasons. First, prostatic abscesses often require surgical drainage, because medical treatment alone can fail and result in relapse.^2^^,^^22^^,^^23^ Second, it emphasizes that urine or prostatic sampling is indicated in any suspected male dog. In humans, prostatic melioidosis correlates with high bacteremia and relapse rates.^22^^,^^23^ Because no neutered males in our study had prostatitis, castration should be considered in infected dogs to prevent the prostate from becoming a nidus for relapse, aligning with standard protocols for other cases of bacterial prostatitis.^25^

The 100% mortality in the septic shock group, compared with the 100% survival in the non-septic SIRS group, provides a prognostic baseline. This finding is similar to the mortality rate of up to 90% reported in humans with septicemic melioidosis in certain regions.^26^ Sepsis is a major determinant of poor outcome in humans, conferring a 3.5-fold increased risk of mortality despite appropriate antimicrobial treatment.^27^ Septic shock was found to be a strong predictor of death in a recent study of melioidosis in humans, with a reported in-hospital mortality adjusted odds ratio of 29.14 (95% CI, 12.45-68.20; ^28^), with similar findings reported in a large case series from Singapore where septic shock requiring inotropic support was significantly associated with inpatient mortality on multivariate analysis.^22^ In our study, the mortality rate of 57% for dogs with sepsis but without shock is comparable to outcomes in humans with melioidosis. Based on the included dogs, the prognosis appears good for those without sepsis, with or without SIRS, with 100% of such dogs surviving long-term. The overall mortality rate of 47% in our study is higher than that reported in the largest prospective case series of melioidosis in humans but, in that case series, the overall mortality rate decreased from 30% in the first 5 years to 9% in the final 5 years.^20^ A similar phenomenon of decreased mortality over time was reported in a second case series, where reported mortality rate between 1989 and 1996 was 40%, but decreased to 16% between 1998 and 2007.^4^ The initial higher overall mortality rates are comparable to the high rate seen in our study. Improved survival in human cases was attributed to earlier diagnosis because of increased awareness among practitioners, earlier institution of treatment protocols including ceftazidime or meropenem, and improvements in intensive care management.^4^^,^^20^ The rapid progression from SIRS to sepsis highlights a window of opportunity for effective treatment. As mentioned, the decrease in mortality rates in humans from approximately 40% to <10% in some regions was achieved by early goal-directed treatment and immediate use of meropenem or ceftazidime. The high mortality in our septic cohort might be at least partially associated with initial use of amoxicillin-clavulanate or fluoroquinolones during the first 48-72 hours, before culture results became available. Our findings support empirical use of ceftazidime or meropenem in septic dogs in endemic areas while awaiting culture results, particularly if a prostatic or cutaneous focus of infection is identified.

Because *B pseudomallei* is a Biosafety Level 3 pathogen, routine antimicrobial susceptibility testing is often unavailable in veterinary practice. However, the 2024 Darwin guidelines note that isolates share a uniform susceptibility profile and acquired resistance is rare, meaning standard empirical recommendations are highly reliable.^29^ In humans, antimicrobial susceptibility testing is reserved for disease recrudescence or relapse. Without antimicrobial susceptibility testing or disease awareness, low initial suspicion frequently results in suboptimal empirical treatment using amoxicillin-clavulanate with or without fluoroquinolones. Data from humans show high *B pseudomallei* susceptibility to imipenem (100%), ceftazidime (99%), doxycycline (94%-99%), amoxicillin–clavulanate (94%-98%), and chloramphenicol (94%-96%; 1,4). Guidelines for humans recommend an intensive phase (IV meropenem or ceftazidime for 10-14 days, extended to 4-6 weeks for severe or deep-seated infections), followed by PO TMPS for 3-6 months.^29^ Trials indicate that initial use of ceftazidime decreases mortality by up to 50% compared with historical regimens.^30^^,^^31^ Furthermore, although overall mortality is similar, amoxicillin-clavulanate results in a significantly higher 72-hour therapeutic failure rate than observed with ceftazidime (25% vs 5%; 32). Ceftazidime is the firstline choice,^29–32^ and fluoroquinolones are not recommended because of intrinsic resistance.^2^ In our cohort, initial empirical choices were suboptimal and preceded culture results, with 17/18 cases diverging from guidelines used in humans. The most frequent initial choices were amoxicillin-clavulanate (14/18) and fluoroquinolones (13/18). After culture, treatment was modified in only 50% (7/14) of surviving dogs: 6/14 were changed to TMPS, and only 3/14 received ceftazidime or meropenem. No dog completed the recommended 14-day IV course, likely because of cost and hospitalization constraints. Despite the lack of veterinary-specific breakpoints, isolating *B pseudomallei* should immediately prompt clinicians to transition to empirically effective intensive and eradication phase treatments.

After the initial intensive phase, adherence to an adequate eradication phase is required to minimize the risks of treatment failure and recurrence.^29^ In humans, the most important determinants of relapse are type and duration of antimicrobial treatment, with relapse risk decreasing by 90% in those who adhere to an appropriate regimen for 12-16 weeks rather than 8 weeks, and relapses are commonly associated with poor antimicrobial compliance.^33^^,^^34^ Oral TMPS monotherapy is recommended for the eradication phase in humans, with other options such as amoxicillin-clavulanate and doxycycline considered less effective.^29^ Only 5/12 of the included animals that were discharged from the hospital were prescribed TMPS for the eradication phase, with an additional 5/12 discharged with amoxicillin-clavulanate, and 2/12 discharged with doxycycline or clindamycin. Of the survivors, median treatment duration was 16 weeks. Of the included animals, 10/18 were discharged and survived > 4 weeks after discharge (with >6 months follow-up available in 9 of these), with only 1/10 euthanized because of disease recurrence. The animal that was euthanized because of recurrence was a cat with osteomyelitis initially treated with clindamycin followed by doxycycline. The other 9/10 dogs (90%) had no recurrence of signs associated with melioidosis. Notably, all 5 dogs discharged on TMPS had no clinical recurrence during the follow-up period. Although it has been suggested that amoxicillin-clavulanate might be a suitable alternative to TMPS for the eradication phase, our findings are in agreement with recommendations in humans that TMPS be considered the first-line antimicrobial for the eradication phase in dogs and cats.

Although only 3 cats were included, the finding that 2/3 (66%) presented with osteomyelitis is notable. In humans, musculoskeletal melioidosis is relatively uncommon, occurring in approximately 10% of cases.^20^ The finding that 2/3 cats in our series and 1/6 in the previous reports suggests *B pseudomallei* has an affinity for bone and joint tissues in cats. Veterinarians should include melioidosis in the differential diagnosis for osteomyelitis or septic arthritis in cats, particularly those in endemic regions or those not responding to standard treatment. Of the 2/3 included cats tested for FIV and FeLV, both were negative and, in humans, pre-existing human immunodeficiency virus infection is not considered a risk factor for development of melioidosis.^2^

The retrospective nature of our study introduced inherent limitations, including lack of standardized diagnostic and treatment protocols. The emergency and referral hospital-based setting might have introduced selection bias toward more severe or complex cases. Additionally, the small sample size for cats precludes definitive conclusions regarding species-specific manifestations, although the presence of osteomyelitis and pyelonephritis suggests a similarly disseminated disease pattern to that seen in dogs and humans.

From a One Health perspective, the emergence of *B pseudomallei* in pets serves as a sentinel for environmental risk. The recent CDC declaration of parts of the United States as endemic regions emphasizes that melioidosis is no longer restricted to traditional tropical hotspots.^2^^,^^6^^,^^7^ Veterinarians working in endemic regions should be aware of the zoonotic potential of this bacterium and the risk it might pose to human companions and clinical staff. Early recognition of potentially compatible clinical signs should prompt the immediate use of appropriate personal protective equipment when dealing with suspected and confirmed cases. In human medicine, the risk of transmission from an infected human to healthcare workers is considered low and although person-to-person transmission is considered very rare, it can occur as a consequence of direct contact with infected bodily fluids or lesions.^35^ A cluster of 5 cases of cutaneous melioidosis in humans occurred in Australia because of the repeated use of contaminated saline used for wound irrigation, demonstrating the ability of the bacterium to survive in bottles and vials of medical fluids.^36^ In accordance with the recommendations in human medicine,^37^ veterinary clinical staff dealing with suspected or confirmed cases of melioidosis should wear gloves, gowns, and a surgical mask when in contact with dogs, fluids, and samples, and strict hand hygiene must be followed. Standard precautions must be taken to avoid needle stick injuries, and it would appear prudent not to reuse any bottles or vials of medication used when treating a confirmed or suspected case. Globally, veterinarians should consider this pathogen in any dog presenting with cutaneous abscessation or open wounds, dogs with prostatitis, cats with osteomyelitis, or any dog with unexplained sepsis or abscessation of internal organs. Early recognition, initial treatment with appropriate antimicrobials such as meropenem or ceftazidime, and a prolonged eradication phase with TMPS might be important to improve survival rates.

## Supplementary Material

Supplementary_Item_1_aalag221

## Off-label antimicrobial declaration

Authors declare no off-label use of antimicrobials.

## Institutional animal care and use committee or other approval declaration

Approved by Mars Veterinary Health Veterinary Clinical Studies Committee.

## Abbreviations

CDC: Centers for Disease Control and Prevention
CT: computed tomography
FeLV: feline leukemia virus
FIV: feline immunodeficiency virus
SIRS: systemic inflammatory response syndrome
TMPS: trimethoprim-sulfonamide
